# Potential of Naturally Derived Alkaloids as Multi-Targeted Therapeutic Agents for Neurodegenerative Diseases

**DOI:** 10.3390/molecules26030728

**Published:** 2021-01-30

**Authors:** Yew Rong Kong, Kai Ching Tay, Yi Xiang Su, Choon Kwang Wong, Wen Nee Tan, Kooi Yeong Khaw

**Affiliations:** 1 Biofunctional Molecule Exploratory Research Group (BMEX), School of Pharmacy, Monash University Malaysia, Jalan Lagoon Selatan, Bandar Sunway 47500, Malaysia; ykon0007@student.monash.edu (Y.R.K.); ktay0002@student.monash.edu (K.C.T.); yxsu1@student.monash.edu (Y.X.S.); cwon0012@student.monash.edu (C.K.W.); 2 Chemistry Section, School of Distance Education, Universiti Sains Malaysia, Penang 11800, Malaysia

**Keywords:** alkaloids, multi-targeted agent, cholinesterase, neuroprotective, neuroinflammation, neurogenesis, amyloid beta, tau protein, drug likeness

## Abstract

Alkaloids are a class of secondary metabolites that can be derived from plants, fungi and marine sponges. They are widely known as a continuous source of medicine for the management of chronic disease including cancer, diabetes and neurodegenerative diseases. For example, galanthamine and huperzine A are alkaloid derivatives currently being used for the symptomatic management of neurodegenerative disease. The etiology of neurodegenerative diseases is polygenic and multifactorial including but not limited to inflammation, oxidative stress and protein aggregation. Therefore, natural-product-based alkaloids with polypharmacology modulation properties are potentially useful for further drug development or, to a lesser extent, as nutraceuticals to manage neurodegeneration. This review aims to discuss and summarise recent developments in relation to naturally derived alkaloids for neurodegenerative diseases.

## 1. Introduction

Neurodegenerative diseases are classified as a group of chronic diseases that are mostly incurable and are characterised by a progressive memory loss and/or neuronal cell death in the central nervous system. Some examples include Alzheimer’s disease (AD) and Parkinson’s disease (PD) and the management plans for these diseases are merely symptomatic treatments that do not halt the disease progression [1,2]. Most of these diseases are closely related to age. Each neurodegenerative disease is associated with diverse clinical manifestations such as cognitive dysfunction and impaired daily functioning [3]. In 2016, there were around 43.8 million people suffering from dementia and 6.1 million people suffering from Parkinson’s disease and the numbers continue to increase annually [4,5]. AD accounts for approximately 75% of all cases of dementia, therefore it is deemed the most common form of dementia [6,7].

AD symptoms include memory loss, and it can progress to an advanced stage that manifests as agitation, apathy, aggression, hallucination, false beliefs and cognitive dysfunction; eventually severe AD patients die from loss of basic physiological functions and complications from infections [8,9,10]. The treatment options for AD are limited to cholinesterase inhibitors including donepezil, rivastigmine and galanthamine, and an *N*-methyl-D-aspartate receptor (NMDA) antagonist named memantine. Whilst cholinesterase inhibitors inhibiting the breakdown of acetylcholine to increase cholinergic neuronal activity in the central nervous system, memantine hindering neurotoxicity induced by excess glutamate [11]. PD is characterised by the presence of slowness of movement and at least one of the following symptoms including resting tremor, postural instability or muscle rigidity. PD is generally managed with medications that increase dopaminergic nerve activity including levodopa and dopamine agonists, as well as medications that suppress dopamine metabolism including catechol-O-methyltransferase (COMT) inhibitors and monoamine oxidase B (MAO-B) inhibitors [12]. Both conditions have no cure that either halts the disease progression or reverses the damage. AD is a multifactorial disease as its etiology is associated with accumulation of amyloid beta, hyperphosphorylation of tau protein, excitotoxicity, oxidative stress and neuroinflammation [7,13]. While, PD is associated with a buildup of intracellular aggregate-containing proteins such as ubiquitin and alpha-synuclein (α-Syn) that form Lewy bodies, dopaminergic neurodegeneration in the substantia nigra pars compacta (SNpc) and neuroinflammation [12,14]. Another similarity of AD and PD is that both diseases are related to neuroinflammation. Neuroinflammation induced by amyloid beta plaques in AD and alpha-synuclein aggregates in PD substantially worsens the loss of cholinergic and dopaminergic neurons, respectively [11,14].

Since the current treatment options solely provide symptomatic relief, ongoing research has been focusing on identifying multi-targeted therapeutic options for neurodegenerative diseases. Natural products are a prolific source of therapeutic leads. For instance, naturally derived alkaloids including huperzine A and galanthamine from medicinal plants have been discovered and explored for their potential in the management of AD [15]. To date, a paradigm shift towards a multi-targeted modulation approach in the management of neurodegenerative diseases is in line with the prevalent characteristic natural product derivatives with multi-targeting properties. This review provides an update on the recent literature on alkaloid-based cholinesterase inhibitors and their *in vivo* efficacy in animal models, highlighting their mechanisms in neurodegenerative-disorder-related experimental models including neuroprotection, neuroinflammation, neurogenesis, tau pathology and amyloid beta accumulation. One of the hurdles in drug development includes drug-like properties. Therefore, an analysis of the physicochemical properties of alkaloid-based compounds is also conducted.

## 2. Cholinesterase Inhibitory Potential of Natural-Product-Derived Alkaloids

**Figure 1:**
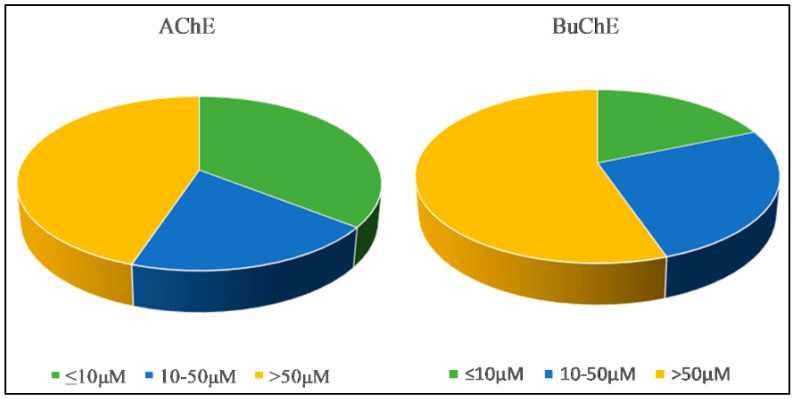
Cholinesterase enzyme inhibition of alkaloids (2011–2020).

Acetylcholinesterase enzyme (AChE) is predominantly found in the cholinergic synapses while butyrylcholinesterase enzyme (BuChE) is a non-substrate specific enzyme that can be found throughout the body including in glial cells. Low levels of AChE and high levels of BuChE have been reported as AD progresses [16]. Acetylcholinesterase enzyme inhibitors (AChEi) including galanthamine (**1**) and donepezil are drugs that have been approved by the Food and Drug Agency (FDA) to manage AD. AChEi is used to enhance acetylcholine (ACh), a neurotransmitter responsible for cognition at a homeostatic level in the brain [17,18,19]. Therefore, chemical compounds able to inhibit AChE enzyme or both AChE and BuChE enzymes (dual inhibitor) are considered essential in the management of the progression of AD. In this review, all naturally occurring alkaloids are categorized by their inhibitory activities in IC_50_. By definition, IC_50_ is a measure of the potency that one chemical substance has to inhibit a specific biological or biochemical function [11]. The inhibition in three categories (IC_50_ ≤ 10 μM, 10–50 μM and >50 μM) is presented in Figure 1.

**Figure 2:**
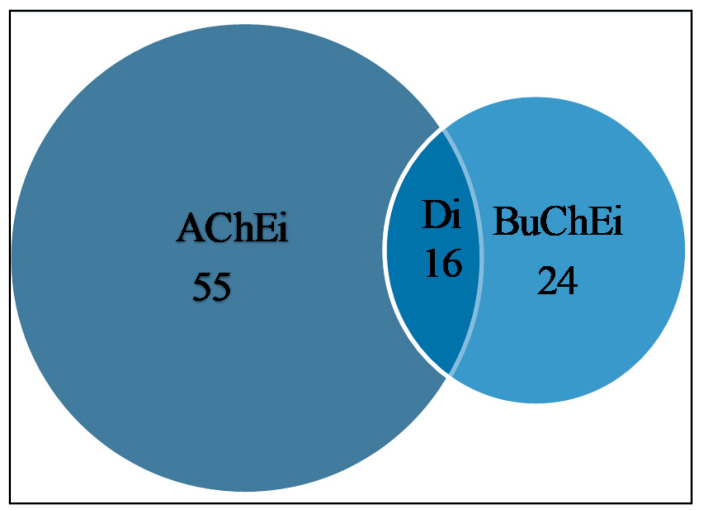
Potential cholinesterase enzyme inhibitors with IC_50_ ≤ 10 μM. AChEi represents acetylcholinesterase inhibitors, Di represents dual cholinesterase inhibitors, BuChEi represents butyrylcholinesterase inhibitors.

**Figure 3:**
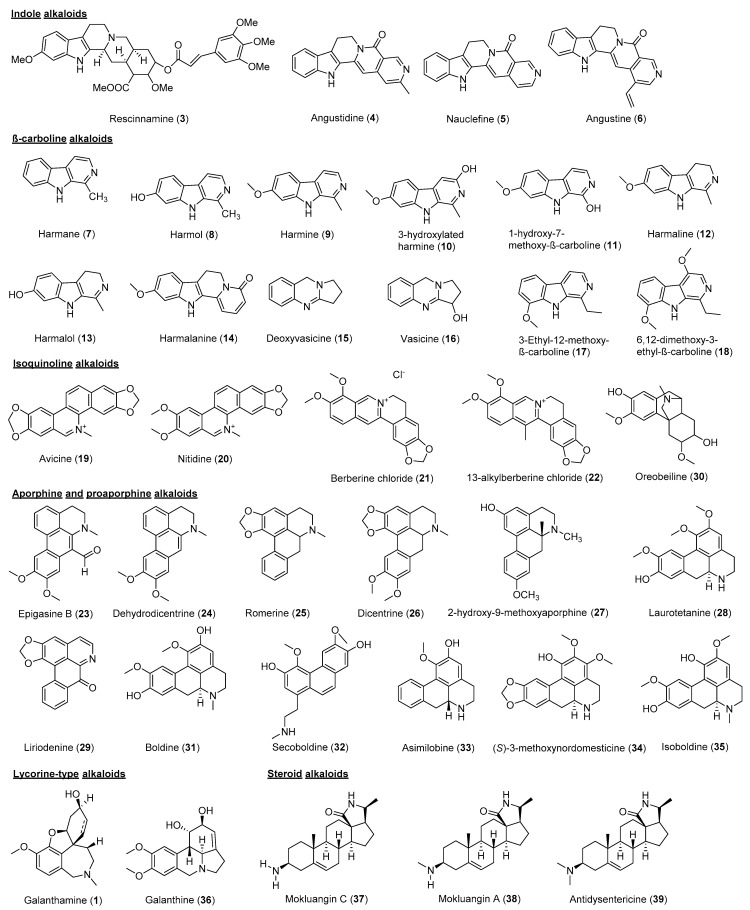
Chemical structures of alkaloids **1**, **3**–**39**.

**Figure 4:**
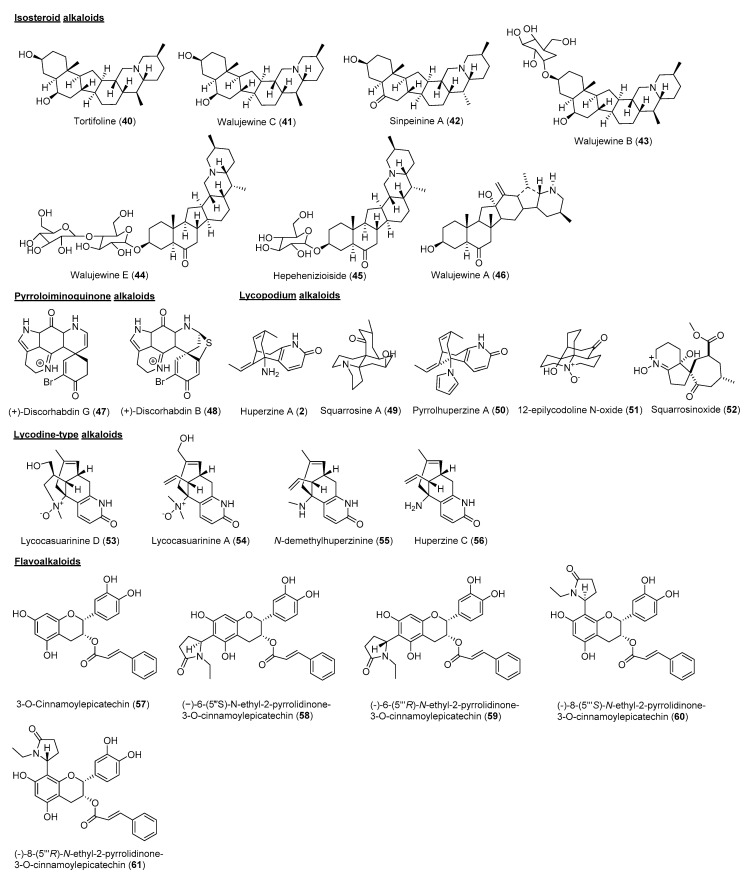
Chemical structures of alkaloid **2**, **40**–**61**.

Figure 2 reveals that a total of 55 alkaloids were identified as AChEi, while 24 alkaloids were identified as BuChEi. Essentially, 16 alkaloids were shown to inhibit both enzymes at IC_50_ values equal to or less than 10 μM. Table 1 lists the bioactive alkaloids isolated from plants along with their cholinesterase inhibitory activities, while Figure 3 and Figure 4 display the chemical structures of alkaloids **1**–**61**.

Indole alkaloids are among the largest group of heterogeneous secondary metabolites, comprising a six-membered aromatic ring fused to a five-membered nitrogen-containing pyrrole ring [36]. Rescinnamine (**3**) from *Rauvolfia reflexa* was reported to behave as a dual cholinesterase inhibitor (IC_50_ AChE 11.01 μM; BuChE 8.06 μM) [20]. Monoterpene indole alkaloids are recognised as molecules containing a monoterpenoid unit fused to an indole moiety. Monoterpene indole alkaloids (**4**–**6**) isolated from the *Nauclea officinalis* possess selective BuChE inhibitory activity. Angustidine (**4**), nauclefine (**5**) and angustine (**6**) were proven to inhibit BuChE with an IC_50_ in the range 1.03 to 7.70 μM. A kinetic study showed that **4** was a mixed-mode inhibitor with a K_i_ value of 6.12 μM [21].

β-carboline alkaloids constitute an indole moiety fused to C-3 and C-4 of a pyridine at its ortho-position [37]. Ten β-carboline alkaloids (**7**–**16**) of *Peganum harmala* were reported to possess cholinesterase inhibitory activities with IC_50_ values <10 μM. Harmol (**8**), harmalol (**13**), deoxyvasicine (**15**) and vasicine (**16**) were potent inhibitors of BuChE with IC_50_ values in the range 0.04 to 0.66 μM. However, harmine was the most potent AChE inhibitor with an IC_50_ of 1.21 μM among the indole alkaloids. A preliminary structure-activity relationship study showed that multiple substitutions at the indole ring and saturation of the pyridine ring were essential for the cholinesterase inhibition effects [22]. 3-Ethyl-12-methoxy-β-carboline (**17**) and 6,12-dimethoxy-3-ethyl-β-carboline (**18**) from *Picrasma quassioides* were reported as possessing AChE inhibitory properties [23].

Isoquinoline alkaloids are among the various classes of alkaloids obtained from natural sources. They consist of an isoquinoline or a tetrahydroisoquinoline ring as their basic skeleton [38]. Avicine (**19**) was the most potent dual cholinesterase inhibitor with IC_50_ values of 0.15 and 0.88 μM for both AChE and BuChE. Nitidine (**20**) had weaker inhibitory activity towards both AChE and BuChE than **19** with IC_50_ values of 0.65 μM and 5.73 μM, respectively [24]. By comparison of the molecular structures, a tertiary amine is present at position 7 of **19** and **20** and is responsible for the high binding affinity towards both AChE and BuChE. Berberine chloride (**21**) and 13-alkylberberine (**22**) are another two isoquinoline alkaloids derived from rhizomes of *Coptis chinensis* that possess slightly different molecular structures from **19** and **20**. Although both **21** and **22** had IC_50_ > 10 μM towards BuChE, **21** displayed a stronger inhibitory activity towards AChE with an IC_50_ of 1.1 μM than **22** with an IC_50_ of 5.6 μM [25]. By comparing the molecular structures of both alkaloids, it is seen that the presence of a methyl group at position 13 in the structure of **22** is responsible for the reduced inhibitory activity against both AChE and BuChE.

The aporphine and proaporphine alkaloids are naturally derived from isoquinoline. Generally, they are distributed in the families of Annonaceae, Lauraceae, Magnoliaceae and Menispermaceae [39]. Examination of the extracts from *Stephania epigaea* [26], *Illigera aromatica* [40], *Beilschmiedia* sp. [27], Monimiaceae and Magnoliacea [41] resulted in the isolation of a series of aporphine and proaporphine-type alkaloids. Among them, epigasine B (**23**), dehydrodicentrine (**24**), romerine (**25**), dicentrine (**26**), 2-hydroxy-9-methoxyaporphine (**27**), laurotetanine (**28**), liriodenine (**29**), oreobeiline (**30**), boldine (**31**), secoboldine (**32**), asimilobine (**33**), (*S*)-3-methoxynordomesticine (**34**) and isoboldine (**35**) were reported to exhibit significant AChE inhibitory activities [26,27].

Lycorine-type alkaloids, also known as Amaryllidaceae alkaloids, belong to the large group of isoquinoline alkaloids. They can be found in plants of the family Amaryllidaceae. Many lycorine-type alkaloids have been isolated, mostly concentrated in bulbs and leaves [42]. Lycorine-type alkaloids including galanthine (**36**) and **1** from *Zephyranthes carinata* showed AChE inhibitory activity against AChE [28].

Steroidal alkaloids are one of the important classes of alkaloids derived from plants. They have a basic steroidal backbone with a nitrogen atom present in the ring or side chain [43]. Three new steroidal alkaloids Mokluangins A–C (**37–39**) from *Holarrhena pubescens* were reported to possess AChE inhibitory activity in the range 1.44 to 4.09 µM, in which substitution at C-3 serves as key in the modulation of AChE inhibitory activity [29].

Isosteroidal alkaloids are one of the representative steroidal alkaloids belonging to the C-27 skeleton type [43]. Seven isosteroidal alkaloids (**40–46**) from *Fritillaria walujewii* were reported for their potential cholinesterase inhibitory activities. Tortifoline (**40**), Walujewine C (**41**), Sinpeinine A (**42**), Walujewine A (**46**), and Walujewine E (**44**) were shown to inhibit AChE with IC_50_ values of 5.8 to 9.8 µM, while all compounds were proven to inhibit BuChE less than 10 µM except **46**. It can be deduced that all compounds are dual cholinesterase inhibitors except **43, 45** and **46** [30].

Pyrroloiminoquinone alkaloids are mainly isolated from marine organisms. Important pyrroloiminoquinone alkaloids include discorhabdins, prianosins, batzellins, wakayins and damirones [44]. Discorhabdins G (**47**) and B (**48**) from Antarctic *Latrunculia* sp. sponges exhibited AChE inhibitory activities with IC_50_ values of 1.3 and 5.7 µM, respectively [31].

Lycopodium alkaloids are an interesting class of alkaloids, commonly found in the plants of Lycopodiaceae. They consist of quinolizine, pyridine and α-pyridone type alkaloids [45]. Generally, lycopodium alkaloids are composed of C_16_ skeletons and occasionally have C_32_ skeletons when they exist as dimers [46]. Among the isolated lycopodium alkaloids, huperzine A (**2**) has appeared as a well-known AChE inhibitor in the treatment of AD [45]. Squarrosine A (**49**) and pyrrolhuperzine A (**50**) were isolated as new Lycopodium alkaloids from *Huperzia squarrosa*, along with known **2** and 12-epilycodoline N-oxide (**51**). Based on the findings, huperzine A was the most potent for AChE inhibition, followed by **49**, **50** and **51** [32]. In a continuation study on the same plant, *H. squarrosa* yielded **52** as a new lycopodium alkaloid with AChE inhibitory potential [33].

Lycodine-type alkaloids belong to the class of lycopodium alkaloids. Generally, they consist of four rings, including one pyridine or pyridone ring. Lycodine-type alkaloids have been known to show AChE inhibitory activity [46]. In a study conducted on lycodine-type alkaloids including Lycocasuarinine D (**53**), Lycocasuarinine A (**54**), *N*-demethylhuperzinine (**55**), and huperzine C (**56**) from *Lycopodiastrum casuarinoides*, they were reported for their cholinesterase inhibition potential. **50** and **51** showed AChE inhibition with IC_50_ values of 0.22 and 4.74 μM, while **55** and **56** were dual cholinesterase inhibitors [34].

Flavoalkaloids are a unique subclass of alkaloids consisting of a basic skeleton of flavonoid fused with a nitrogen containing ring, such as pyrrolidinone, pyrrolidine, indole, piperidine, piperidinone and aminoglycoside [47]. To date, less than 100 naturally occurring flavoalkaloids have been reported, although they have been found to show a wide range of bioactivities [35,47]. In a recent study, five new cinnamoylated flavoalkaloids were isolated from *Camellia sinensis*. The compounds were known as 3-*O*-cinnamoylepicatechin (**57**), (−)-6-(5‴*S*)-*N*-ethyl-2-pyrrolidinone-3-*O*-cinnamoylepicatechin (**58**), (−)-6-(5‴*R*)-*N*-ethyl-2-pyrrolidinone-3-*O*-cinnamoylepicatechin (**59**), (−)-8-(5‴*S*)-*N*-ethyl-2-pyrrolidinone-3-*O*-cinnamoylepicatechin (**60**) and (−)-8-(5‴*R*)-*N*-ethyl-2-pyrrolidinone-3-*O*-cinnamoylepicatechin (**61**). All flavoalkaloids showed significant AChE inhibitory activity with IC_50_ ranging from 0.126 to 1.040 μM. The study revealed that the C-6 position was crucial in AChE inhibitory activities [35].

## 3. Multi-Target Modulation Potential of Alkaloids in Neurodegenerative Diseases

For decades, researchers have been heavily focused on discovering drugs that can potentially modify the progression of neurodegenerative diseases, for example, the discovery of secretase inhibitors based on the amyloid hypothesis for Alzheimer’s disease. Unfortunately, none of the candidates have shown promising results improving the progressing of the disease in the final phase of clinical trials. Cholinesterase inhibitors have remained the drugs for the management of the disease and further studies are prompted to explore the potential of alkaloids for the management of neurodegenerative conditions in addition to their cholinesterase activity [48]. Despite having potent anti-cholinesterase properties that help to improve cognitive function, we have conducted a search on the alkaloids in Table 1 that have potential cholinesterase inhibitory activities and compiled several studies that have reported the potential of these alkaloids to act on multiple other targets, to provide an overview of the multi-target modulation potential of these compounds. However, it cannot be ruled out that compounds with lesser cholinesterase inhibitory activities are not potentially useful for some other targets in neurodegenerative diseases. This section focuses mainly on Alzheimer’s disease (AD) and Parkinson’s disease (PD). The collection of mechanisms that these alkaloids act upon include neuroprotection, neuroinflammation, neurogenesis, amyloid beta aggregation (Aβ) and tau hyperphosphorylation.

### 3.1. Neuroprotection

Neuroprotection slows down loss of neurons and subsequently progression of neurodegenerative diseases via various pathways, including reduction in oxidative stress, mitochondrial dysfunction, protein aggregation, inflammation, excitotoxicity and cell apoptosis [49].

Several studies have shown that harmane (**7**), harmol (**8**), harmine (**9**), harmaline (**12**), and harmalol (**13**) which belong to the indole β-carboline class (Table 1), exhibit neuroprotective potential against neuronal damage. An *In Vitro* study showed that **7** protects against H_2_O_2_-induced toxicity in neuroblastoma cells by attenuating the decreased cell viability [50]. This model is associated with neuronal injuries caused by oxidative stress that is implicated in neurodegenerative disease development.

**12** is widely used to induce tremor in rodents, no negative impact was found on dopaminergic PC12 cells alone at concentration of 50 µM, same for its metabolite harmalol **13** [51]. The result coincides with two other studies that showed no toxicity imposed on PC12 cells by **12** and **13** alone [52,53]. MPP+ is a neurotoxin to the dopaminergic neurons that implicated in Parkinson’s disease (PD). The role of **7** in suppressing 1-Methyl-4-phenylpyridinium (MPP+) effects is achieved by suppressed mitochondrial transmembrane potential (MMP), cytochrome c release, caspase-3 activation, reactive oxygen species (ROS) and GSH levels *In Vitro* [51,52,53]. Dopamine oxidation initiates different cascades to form endogenous neurotoxins that contribute to neurodegeneration [54,55]. In the case of **12**, it was shown to offset the toxic effects of dopamine oxidation imposed on brain mitochondria *Ex Vivo*. This is attributed to the antioxidative properties of **12** via maintaining thioredoxin reductase activity and inhibiting thiol oxidation and therefore dopamine oxidation product formation [56]. In addition, **9** and **12** upregulated antioxidant enzymes such as superoxide dismutase [57] and glutathione peroxidase (GPx) *In Vitro*, while in other studies, they reduced ROS elevation and thiol oxidation, leading to an enhanced antioxidant defence mechanism to produce a neuroprotective effect [52,53,58,59].

In addition, **9** exerted neuroprotective effects by upregulating glutamate transporter-1 (GLT-1) protein levels and GLT-1 and glutamate aspartate transporter (GLAST)-dependent glutamate uptake in astroglial cells and in the cortical tissue of SOD1^G93A^ mice, a transgenic mouse model of amyotrophic lateral sclerosis [60]. These glutamate transporters maintain low extracellular concentration of glutamate, which is an excitatory neurotransmitter, whereby its accumulation contributes to excitotoxicity [61]. Moreover, a recent systematic review showed that **9** improved memory and learning and demonstrated neuroprotective effects on the hippocampus in preclinical experimental models [62]. It was purportedly involved in GLT-1 upregulation, ROS decrement, brain-derived neurotrophic factor (BDNF) elevation and had anti-inflammatory effects. In the context of PD, **9** was recently investigated for its degradation effect on α-synuclein *In Vitro*. Ubiquitin-proteasome system (UPS) is one of the systems that removes α- synuclein via proteasome proteolytic activity and it was shown that **9** increased the proteolytic activity via protein kinase A (PKA) phosphorylation and hence enhanced UPS for α-synuclein clearance [63,64]. 

**13**, a metabolite of **9**, was included in the results from previously mentioned studies as a compound of interest and it was found that **13** provided neuroprotection via modulating oxidative stress, MMP and apoptosis *In Vitro* [51,52,53]. These findings showed a decrease in ROS and thiol oxidation, an increase in GSH levels and attenuation in MMP loss [51,52,53,65]. The effect of **13** on two of the processes involved in mediating cell death, which are release of cytochrome c and caspase-3 activation, were investigated as well, and **13** decreased both [51,66].

Research on the protective effects of berberine chloride (**21**), which is an isoquinoline alkaloid (Table 1), on neurodegeneration has been gaining popularity in recent years. In an *in vivo* study, pretreatment of **21** on 6-hydroxydopamine (6-OHDA)-stimulated neurotoxicity that models PD in rats significantly reduced apomorphine-induced rotations and the loss of Nissl-stained substantia nigra pars compacta (SNpc) neurons, and attenuated the reduction of tyrosine hydroxylase immunoreactivity in SNpc dopaminergic neurons [67]. An *In Vitro* study showed that **21** exerted neuroprotective properties against oxidative-stress-mediated neurodegeneration through activating Akt/GSK, Akt/GSK3β/Nrf-2 and PI3K/Akt/Nrf2 signalling cascades, increasing p-CREB, stimulating NGF and BDNF release, decreasing NF-κB nuclear translocation, suppressing TNFα, COX-2, IL-1B, NF-κB, and downregulating caspase-1, caspase-3, Bax, Bcl-2 elevation, cyclin D1, and p53 [68]. Aside from the results on par with previous studies, Deng and the team reported **21** decreased ROS production and reversed MMP reduction *In Vitro*, and suggested **21** exerted neuroprotective effects by PI3K/Akt pathway activation in rotenone-induced neurotoxicity [69]. **21** was also proven to protect PC-12 cells from oxidative damage via PI3K/AKT/mTOR-mediated mitophagy [70]. Wallerian-like degeneration (WLD) is an axonal degeneration, extending from the axon distal to the site of injury and occurs in neurodegenerative diseases [71]. Interestingly, **21** was found to be a non-competitive inhibitor of Sterile Alpha and Toll Interleukin Receptor Motif–containing protein 1 (SARM1), which is a key mediator for WLD, yielding a percentage inhibition from a primary screen of 70% [72]. The same study further tested its inhibition activity on the SAM^1−2^TIR domain of bacterial and Expi293 cells to confirm the findings and obtained IC_50_ values of 110 ± 10 μM and 77 ± 5 μM, respectively. In addition, **21** increased CYP2J2, a protein that was found to be protective against a PD model, via stimulation of Peroxisome proliferator-activated receptor alpha (PPAR-α) *In Vitro* [73]. An *in vivo* study showed that **21** prevented aminolevulinic acid dehydratase (δ-ALA-D) inhibition and prevented damage on purinergic transmission by attenuating NTPDase, ADP, 5′-nucleotidase and ADA activity loss in streptozotocin-induced dementia, in which the regulation of this transmission plays a role in memory processing [74,75]. However, δ-ALA-D activity in specific neuronal cells warrants further investigation due to the limited studies available.

The stimulation of glial hemichannel activity enhances ATP and glutamate release that subsequently induces neuronal death, whereas Reticulon-3 (RTN3) aggregates in the AD brain and facilitates development of dystrophic neurites [76,77]. Boldine (**31**), an aporphine alkaloid (Table 1), was reported to decrease astroglial hemichannel activity without affecting gap junctional communication, reducing ATP and glutamate release. An *in vivo* study showed **31** protected against neuronal oxidative stress and neuritic dystrophies surrounding amyloid beta (Aβ) plaques, where smaller and fewer areas of RTN3 immunoreactive dystrophic neurites (RIDNs) were observed in APP/PS1 mice [78]. In another study, **31** decomposed H_2_O_2_, decreased iron and EDTA-mediated deoxyribose degradation and formation of melanin by dopamine oxidation, attenuated loss of MMP, cytochrome C elevation, loss of thioredoxin reductase activity, inhibited thiol oxidation induced by dopamine and 6-OHDA and PC-12 cell viability loss, and caspase-3 activation induced by dopamine [79].

An *in vivo* study showed that deoxyvasicine (**15**), which is a β-carboline alkaloid (Table 1), protected the neurons from oxidative-stress-induced damage at a dose of 15 mg kg^−1^. The study demonstrated that the amount of glutathione peroxidase (GPx) was significantly elevated by administration of **15**, subsequently enhancing the antioxidant defence mechanism in the brain. In addition, **15** was capable of enhancing the survival of nerves by elevating brain-derived neurotrophic factor (BDNF) at 45 mg kg^−1^ [80]. 

### 3.2. Neuroinflammation

Over the years, studies have been carried out to investigate the pathology of neurodegenerative conditions and neuroinflammation has been found to be one of the underlying mechanisms. A defect in immune cell function can initiate inflammation in the central nervous system (CNS) and eventually cause nerve injury. In particular, acute inflammation can be beneficial to the brain. Conversely, chronic inflammation can harm the brain by promoting hyperphosphorylation of tau protein and amyloid beta (Aβ) aggregation. These mechanisms can be activated by proinflammatory mediators such as interleukin 1 (IL-1) and interleukin 6 (IL-6). Since inflammation is closely related to the pathology of neurodegenerative conditions such as AD and PD, alkaloids that are able to prevent neuroinflammation might be able to ameliorate neuroinflammation as well as helping to manage the conditions [81,82,83].

**9** and **12** were highlighted to be capable of counteracting neuroinflammation. It was suggested that they were able to decrease the production of inflammatory cytokines such as tumour necrosis factor alpha (TNF-α) and myeloperoxidase (MPO) and mediators such as nitric oxide (NO). In particular, **7** and **12** showed IC_50_ of 0.08 μM and 0.26 μM towards MPO, respectively, therefore proving their potential to be incorporated as an agent to counteract neuroinflammation [58,59]. In addition, an *in vivo* study suggested that **9** significantly improved cognitive deficits in chemically induced diabetic rats at 20 mg kg^−1^. The study proposed that diabetes mellitus was closely related to cognitive dysfunction and both were related to inflammation. When **9** was administered to the diabetic rats, NLRP3 inflammasome activity was supressed, leading to an increase in the expression of brain-derived neurotrophic factor (BDNF) and subsequently improved cognitive ability [84]. In addition, an *in vivo* study reported that **9** was also capable of crossing the blood–brain barrier (BBB) soon after oral intake [85].

Another β-carboline alkaloid **15** was able to inhibit neuroinflammation via several pathways. An *in vivo* study had proved that **15** was capable of increasing the amount of γ-GABA and decreasing the amount of glutamate in the brain at concentrations 5 ^1^, 15 and 45 mg kg^−1^. γ-GABA is a vital neurotransmitter that acts to inhibit neuroinflammation initiated by astrocytes and microglia by hindering the release of TNF-α. Besides, production of TNF-α, which is a cytokine that promotes inflammation, was also significantly downregulated in a concentration-dependent pattern when treated with **15** at concentrations of 5–45 mg kg^−1^ [80]. 

**21** acts on multiple targets to improve AD conditions. One of the mechanisms of action of **21** is to attenuate neuroinflammation [86]. For example*,* an *in vivo* study reported a decrease in pro-inflammatory mediators including COX-2, IL-12, IL-6, IL-1β and TNF-α resulting from the administration of **21** in an AD rat brain [87]. Furthermore, an *in vivo* study reported that **21** hindered the p65 subpart expression and phosphorylation at 50 mg kg^−1^. p65 is a subpart of the NF-κB heterodimer that is vital in modulating inflammatory protein production [88]. In addition, an *in vivo* study also reported that **21** was able to counteract neuroinflammation by suppressing IL-6, TNF and p38 MAPK signalling cascades [89]. 

### 3.3. Neurogenesis

Adult neurogenesis is a process that occurs in the subgranular zone (SGZ) and subventricular zone (SVZ) of the hippocampus to produce neurons throughout a human’s lifetime. A defect in adult neurogenesis can potentially result in neurodegenerative conditions such as Alzheimer’s disease (AD), Parkinson’s disease (PD) and Huntington’s disease (HD) [90]. For instance, an *in vivo* study reported that adult neurogenesis impairment may have started in the early AD disease stage even before the formation of neurotoxic neurofibrillary tangles and Aβ plaques [91,92].

An *In Vitro* study suggested that **8**, **9**, and **12** successfully induced neurogenesis when tested on progenitor cells cultured from SGZ and SVZ. Furthermore, the metabolism of **9** in the human body produced **8** as the main product. These β-carboline alkaloids were also capable of inducing neuronal cell specialization and this was proven by the presence of MAP-2 and Tuj-1 expression. The study proposed that the neurogenesis potential of these β-carboline alkaloids was attributed to their inhibitory activity towards monoamine oxidase [70] and DYRK1A [93]. 

An *In Vitro* study investigating the neurogenesis potential of aporphine alkaloids (Table 1) on PC-12 cells showed that asimilobine (**33**) significantly stimulated the outgrowth of neurite in PC-12 cells but with no obvious effect on the mRNA expression coding for proteins essential for cell division and specialisation. In addition, the study also reported that **33** was highly penetrable across the blood–brain barrier (BBB), making it a potential drug candidate for the management of neurodegenerative conditions [94]. 

### 3.4. Aggregation of Amyloid Beta

Amyloid beta (Aβ) is a product resulting from the enzymatic breakdown of amyloid precursor protein (APP) by γ-secretase and β-secretase and it is suggested that Aβ plays a role in the development of AD. Aβ is a peptide that mostly consists of 40–42 amino acids and is susceptible to aggregation to form neurotoxic Aβ plaques. Once Aβ plaques accumulate in the brain, abnormal synaptic and neuronal activities will develop, causing damage to the brain and producing symptoms such as cognitive deficits and gradual loss of memory [95,96]. Therefore, preventing the aggregation of Aβ may help to delay the progression of AD. Aside from the anti-cholinesterase activity displayed by plant-derived alkaloids, some of them are capable of inhibiting Aβ aggregation. 

Avicine (**19**) and nitidine (**20**) are isoquinoline alkaloids (Table 1) and they possess the ability to inhibit oligomerization of Aβ. An *In Vitro* study reported that **19** and **20** demonstrated moderate intensity suppression towards Aβ aggregation as seen from their IC_50_ values of 5.56 and 1.89 μM towards Aβ, respectively. In fact, it was also suggested that both **19** and **20** possessed the most significant inhibition activity towards multiple targets involved in neurodegenerative conditions and they had similar molecular structures when compared to other isoquinoline alkaloids extracted from *Zanthoxylum rigidum*. Therefore, it is encouraged to investigate the molecular scaffold associated with their multi-targeted activity [24].

In addition, **21** has been studied for years and a review reported that it hindered ERK1/2 and mitogen-activated protein kinase (MAPK) signalling cascades, consequently deactivating the β-secretase-1 (BACE-1) and diminishing Aβ generation [86]. In fact, **21** is marketed as an over-the-counter product that is consumed orally in China, as studies investigating its efficacy and safety after oral consumption reported acceptable results. Apart from its anti-cholinesterase activity, which is comparable to **1** and **21** also possessed the ability to decrease Aβ aggregation by decreasing the formation of Aβ. An *in vivo* study reported that **21** was able to significantly inhibit β-secretase activity in an AD brain, consequently reducing the formation of Aβ by up to 40% and improving the symptoms of AD. Aside from suppressive action towards β-secretase, another *in vivo* study reported that **21** enhanced Aβ40 activity that lowered the neurotoxic potential of Aβ42 by modifying the stability, morphology and solubility of Aβ42 to impair Aβ42 aggregation [87,97]. This statement is further supported by an *in vivo* study that claimed that **21** at concentrations of 50 and 100 mg/kg/day was able to downregulate the Pen-2, Aph-1α and PS1 parts of γ-secretase and β-secretase, consequently suppressing the production of Aβ. In addition, **21** was shown to significantly increase α-secretase activity at identical concentrations [98]. An *In Vitro* study further suggested that **21** was able to inhibit oligomerization of Aβ and fibril formation [99].

### 3.5. Tau Hyperphosphorylation

Tau protein (τ) plays a vital role in stabilizing the neuronal microtubules and hence supporting the structure of a neuron and the transport of nutrients intracellularly. Kinases that include dual specificity tyrosine phosphorylation regulated kinase 1A (DYRK1A), glycogen synthase kinase-3β (GSK-3β), Ca^2+^/calmodulin activated protein kinase II and cyclin-dependent kinase-5 (Cdk5) can induce tau hyperphosphorylation. Hyperphosphorylation of tau protein leads to the clumping of phosphorylated tau proteins to form neurofibrillary tangles or paired helical filament tau. Consequently, they no longer support the microtubules within a neuron, eventually leading to neuronal apoptosis and hence neurodegeneration [100].

**9** was found to possess inhibitory activities towards DYRK1A that in turn hindered tau protein hyperphosphorylation. It was proven by an *In Vitro* study that **9** was a strong inhibitor of DYRK1A with an IC_50_ of approximately 80 nM. **9** deactivated DYRK1A, hence suppressing tau phosphorylation on serine 396, subsequently reducing all three types of phosphorylated forms of tau protein. By reducing the amount of phosphorylated tau, harmine (**9**) preserved the function of tau to support the microtubules in a neuron, preventing neuronal death. However, it was reported that **9** could be neurotoxic at concentrations beyond 8 μM due to excessive tau protein depletion [100]. 

Aside from **9**, **21** was also able to impede hyperphosphorylation of tau protein. Although more research needs to be carried out on the actual mechanism of **21** to decrease tau hyperphosphorylation, two *In Vitro* studies have shown that **21** is capable of preventing the hyperphosphorylation of tau protein stimulated by calyculin A at concentrations of 20 and 25 μg mL^−1^ by diminishing GSK-3β activity and upregulating the activity of protein phosphatase 2A. In addition, reversal of tau phosphorylation was induced by **21** at Ser262 [86,101,102]. An *in vivo* study also reported that the antioxidant properties of **21** could help to inhibit overexpression of tau protein and tau hyperphosphorylation in an AD brain [87]. 

## 4. Physicochemical Analysis of the Alkaloids

**Figure 5:**
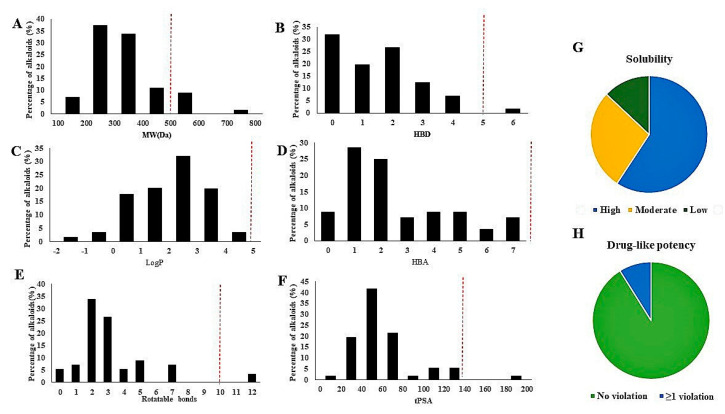
Analysis of physicochemical properties of alkaloids: (**A**) molecular weights (MW(Da)); (**B**) hydrogen bond donors (HBDs); (**C**) calculated LogP; (**D**) hydrogen bond acceptors (HBAs); (**E**) rotatable bonds; (**F**) topological polar surface area (TPSA); (**G**) solubility; (**H**) Drug-like potency.

Figure 5 shows the analysis of the physicochemical properties of the alkaloids included in this review. The molecular weight of the majority compounds was below 500 Da. The higher molecular weight compounds included **44**, **45**, and flavoalkaloids (**58**–**61**). Only one compound violates the Lipinski rule for hydrogen bond donating, rotatable bonds and topological polar surface area (TPSA). Interestingly, all the compounds included in this review followed the Lipinski rule for Log P and hydrogen bond acceptor (HBA). 32 compounds were classified as highly soluble, 15 compounds had moderate solubility and 7 compounds had low solubility.

## 5. Method

All naturally derived alkaloids reported for their cholinesterase inhibitory activity in the literature between 2011–2020 were included in this study. Synthetic alkaloids, alkaloids tentatively identified by Gas chromatography-mass spectrometry (GC-MS) and Liquid chromatography-mass spectrometry LCMS reported for their cholinesterase inhibitory were excluded in this study. Figure 1 shows that 38% and 18.7% of the AChE and BuChE inhibitors with IC_50_ ≤ 10 μM were included in this review. As a result, 61 compounds with IC_50_ less than or equal to 10 μM except **1** and **2** were included in the text and review for the disease-modifying potential including amyloid beta inhibitory, tau hyperphosphorylation inhibitory, neuro-inflammation, neurogenesis and neuroprotective effects. The physicochemical properties of the 59 alkaloids (except galanthamine and huperzine A) were predicted (Instant JChem 17.10.0, 2020 ChemAxon Ltd. (http://www.chemaxon.com)).

## 6. Conclusions

A growing body of evidence has shown the importance of naturally derived alkaloids as neurodegenerative disease modulators. Although alkaloids are the major source of AChE inhibitors, a significant number of dual cholinesterase inhibitors and BuChE inhibitors are being discovered. It is interesting to note that several alkaloids documented in this review protect neurons against mechanisms which are deleterious such as neuroinflammation, oxidative stress, excitotoxicity, apoptosis, Aβ accumulation and tau phosphorylation, and could be developed for the management of Alzheimer’s and Parkinson’s diseases. Specifically, **9** and **21** are potential modulators in the management of the progression of these diseases. Nevertheless, dosage of these alkaloids to be used in neurodegenerative diseases remained inconclusive. Physicochemical analysis revealed that the majority of alkaloids follow the Lipinski rules of drug likeliness. The blood–brain barrier is an important factor guarding the penetration of compounds into the brain, and requires further attention to be paid to it. Understandably, the setbacks of naturally derived compounds such as low extraction yield have halted the development of potential candidates into therapeutic leads. Further development is needed to improve their usefulness as viable therapeutics.

## Figures and Tables

**Table 1:** Alkaloids from different species and respective IC_50_ towards AChE and BuChE.

Species	Part	Alkaloid Class	Chemical Constituent	IC_50_ (μM)		References
				AChE	BuChE	
*Rauvolfia reflexa*	Bark	Indole	Rescinnamine (**3**)	>10	8.06	[20]
*Nauclea officinalis*	Bark	Indole β-carboline	Angustidine (**4**)	>10	1.03	[21]
			Nauclefine (**5**)	-	7.70	
			Angustine (**6**)	-	4.98	
*Peganum harmala*	Seed	Indole β-carboline	Harmane (**7**)	3.64	1.04	[22]
			Harmol (**8**)	1.90	0.35	
			Harmine (**9**)	1.21	2.79	
			3-Hydroxylated harmine (**10**)	>10	3.25	
			1-Hydroxy-7-methoxy-β-carboline (**11**)	7.19	5.15	
			Harmaline (**12**)	1.95	5.38	
			Harmalol (**13**)	3.45	0.66	
			Harmalanine (**14)**	>10	3.24	
		β-carboline	Deoxyvasicine (**15**)	2.37	0.04	
			Vasicine (**16**)	3.38	0.10	
*Picrasma quassioide*	Stem	β-carboline	3-Ethyl-12-methoxy-*β*-carboline (**17**)	6.37	-	[23]
			6,12-Dimethoxy-3-ethyl-*β*-carboline (**18**)	9.01	-	
*Zanthoxylum rigidum*	Root	Isoquinoline	Avicine (**19**)	0.15	0.88	[24]
			Nitidine (**20**)	0.65	5.73	
*Coptis chinensis*	Rhizome	Isoquinoline	Berberine chloride (**21**)	1.1	> 10	[25]
			13-alkylberberine (**22**)	5.6	> 10	
*Stephania epigaea*	Root	Proaporphine Aporphine	Epigasine B (**23**)	4.36	-	[26]
			Dehydrodicentrine (**24**)	2.98	-	
			Romerine (**25**)	8.32	-	
			Dicentrine (**26**)	6.6	-	
*Beilschmiedia alloiophylla* *Beilschmiedia kunstleri*	Bark	Aporphine	2-Hydroxy-9-methoxyaporphine (**27**)	2.0	-	[27]
			Laurotetanine (**28**)	3.2	-	
		Oxoaporphine	Liriodenine (**29**)	3.5	-	
		Morphinan (isoquinoline)	Oreobeiline (**30**)	5.0	-	
		Aporphine	Boldine (**31**)	8.5	-	
			Secoboldine (**32**)	10.0	-	
			Asimilobine (**33**)	8.7	-	
			(*S*)-3-Methoxynordomesticine (**34**)	10.0	-	
			Isoboldine (**35**)	9.4	-	
*Zephyranthes carinata*	Whole plant	Lycorine-type	Galanthine (**36**)	6.10	-	[28]
			Galanthamine (**1**)	1.27	-	
*Holarrhena pubescens*	Bark	Steroid	Mokluangin C (**37**)	1.44	-	[29]
			Mokluangin A (**38**)	2.12	-	
			Antidysentericine (**39**)	4.09	-	
*Fritillaria walujewii*	Bulb	Isosteroid	Tortifoline (**40**)	5.8	2.08	[30]
			Walujewine C (**41**)	7.2	2.58	
			Sinpeinine A (**42**)	8.3	3.05	
			Walujewine B (**43**)	>10	3.89	
			Walujewine E (**44**)	9.8	5.71	
			Hepehenizioiside (**45**)	>10	6.80	
			Walujewine A (**46**)	7.6	>10	
Antarctic *Latrunculia* spp.	Sponge	Pyrroloiminoquinone	(+)-Discorhabdin G (**47**)	1.3	-	[31]
			(+)-Discorhabdin B (**48**)	5.7	-	
*Huperzia squarrosa*	Aerial	Lycopodium	Squarrosine A (**49**)	7.3	-	[32]
			Pyrrolhuperzine A (**50**)	8.91	-	
			12-epilycodoline N-oxide (**51**)	0.59	-	
			Huperzine A (**2**)	0.01	-	
*Huperzia squarrosa*	Whole plant	Lycopodium	Squarrosinoxide (**52**)	3.12	-	[33]
			Huperzine A (**2**)	0.034	-	
*Lycopodiastrum casuarinoides*	Aerial part	Lycodine-type	Lycocasuarinine D (**53**)	0.22	>10	[34]
			Lycocasuarinine A (**54**)	4.74	>10	
			N-demethylhuperzinine (**55**)	0.89	1.86	
			Huperzine C (**56**)	0.37	7.33	
*Camellia sinensisvar*	Leaf	Flavoalkaloid	3-*O*-Cinnamoylepicatechin (**57**)	1.0	-	[35]
			(−)-6-(5‴*S*)-*N*-ethyl-2-pyrrolidinone-3-*O*-cinnamoylepicatechin (**58**)	0.14	-	
			(−)-6-(5‴*R*)-*N*-ethyl-2-pyrrolidinone-3-*O*-cinnamoylepicatechin (**59**)	0.13	-	
			(−)-8-(5‴*S*)-*N*-ethyl-2-pyrrolidinone-3-*O*-cinnamoylepicatechin (**60**)	0.18	-	
			(−)-8-(5‴*R*)-*N*-ethyl-2-pyrrolidinone-3-*O*-cinnamoylepicatechin (**61**)	0.21	-	

## Data Availability

Not applicable.
